# Supplementary material for: A non-invasive far-red light-induced split-Cre recombinase system for controllable genome engineering in mice
Source: Nat Commun. 2020 Jul 24;11:3708. doi: 10.1038/s41467-020-17530-9 (PMC7381682; doi:10.1038/s41467-020-17530-9)
Supplement: Supplementary file 1 — Supplementary Information [file 41467_2020_17530_MOESM1_ESM.pdf]

Supplementary Information

A non-invasive far-red light-induced split-Cre recombinase system for controllable genome engineering in mice

Jiali Wu *et al*.

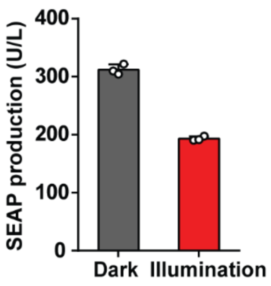

Supplementary Figure 1. SEAP production of a far-red light-induced split Cre-*loxP* system. 6 × 10^4 HEK-293 cells per well were co-transfected with four plasmids: pXY137 (P_{hCMV}-p65-VP64-BldD-pA::P_{hCMV}-BphS-P2A-YhjH-pA, 100 ng), pXY110 (P_{hCMV}-CreN59-L0-Coh2-NES-pA, 100 ng), pXY111 (P_{FRLa}-NLS- DocS-L0-CreC60-pA, 100 ng), pGY125 (P_{hCMV}-*loxP*-STOP-*loxP*-SEAP-pA, 200 ng), and then illuminated for 6 h with FRL (1.5 mW cm^{-2}, 730 nm) each day for two days. SEAP expression in the culture supernatants was profiled at 48 h after the first illumination**.** Data represent the mean ± SD; *n* = 3 independent experiments. Source data is available in the Source Data file.

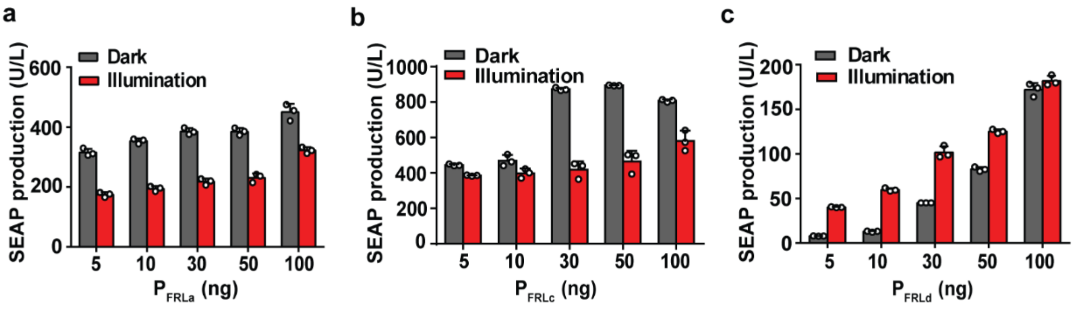

Supplementary Figure 2. Optimization of the different FRL-responsive promoters P_{FRLx} driving DocS-CreC60 expression. 6 × 10^4 HEK-293 cells per well were co-transfected with pXY137 (100 ng), pXY110 (100 ng), pGY125 (SEAP reporter plasmid, 200 ng) and different amounts (5-100 ng) of (**a**) pXY111 (P_{FRLa}-NLS-DocS-L0-CreC60-pA), (**b**) pXY121 (P_{FRLc}-NLS-DocS-L0-CreC60-pA) or (**c**) pXY133 (P_{FRLd}-NLS-DocS-L0-CreC60-pA), and then illuminated for 6 h with FRL (1.5 mW cm^{-2}, 730 nm) each day for two days. SEAP expression in the culture supernatants was profiled at 48 h after the first illumination. All data represent the mean ± SD; *n* = 3 independent experiments. Source data is available in the Source Data file.

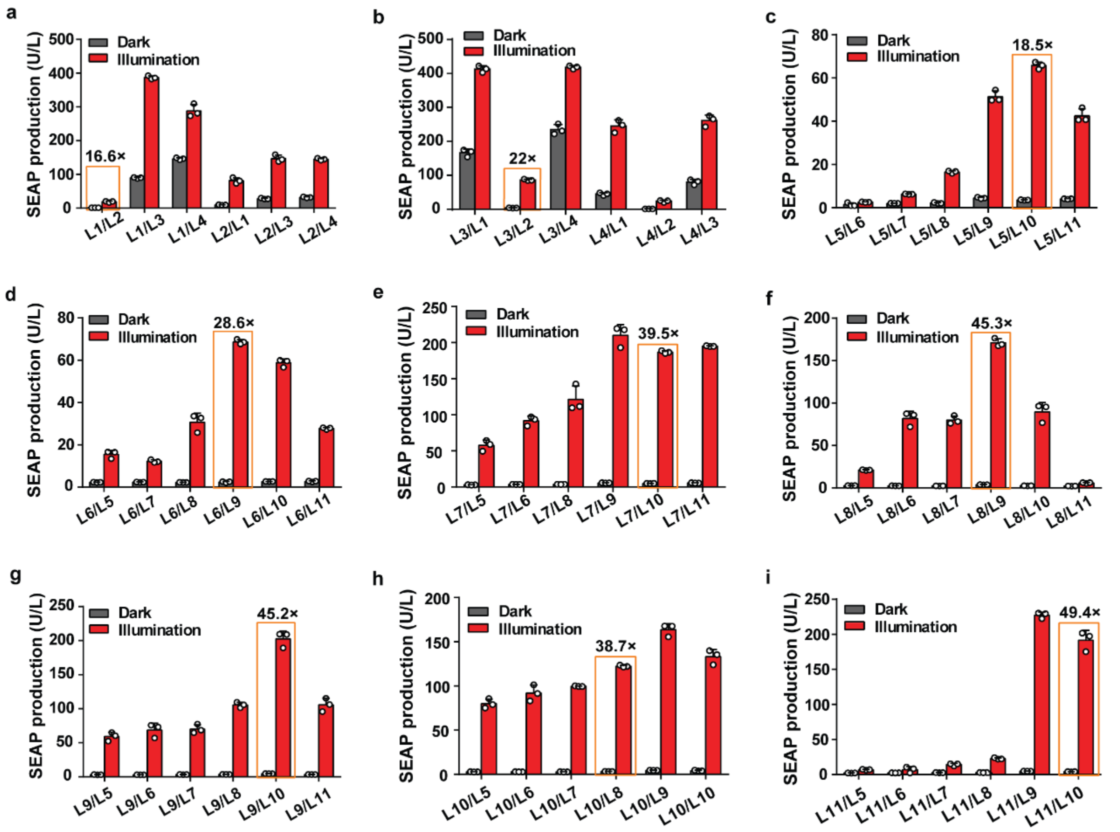

Supplementary Figure 3. Optimization of different combination of the linker amino acid sequences between CreN59 and Coh2 as well as between CreC60 and DocS. Different linker amino acid sequences are listed in **Supplementary Table 1. (a)** linker 1 combined with linker 2-4 and linker 2 combined with linker 1, 3, 4. **(b)** linker 3 combined with linker 1, 2, 4. **(c)** linker 5 combined with linker 6-11. **(d)** linker 6 combined with linker 5, 7, 8, 9, 10, 11. **(e)** linker 7 combined with linker 5, 6, 8, 9, 10, 11. **(f)** linker 8 combined with linker 5, 6, 7, 9, 10, 11. **(g)** linker 9 combined with linker 5, 6, 7, 8, 10, 11. **(h)** linker 10 combined with linker 5, 6, 7, 8, 9, 11. **(i)** linker 11 combined with linker 5-10. 6×10^4 HEK-293 cells were co-transfected with pXY137 (100 ng), pGY125 (200 ng) and different combination of the plasmids encoding CreN59-Coh2 fusion protein or DocS-CreC60 fusion protein with different linkers (10 ng), **Supplementary Table 2**, and then illuminated for 6 h with FRL (1.5 mW cm^{-2}, 730 nm) each day for two days. SEAP expression in the culture supernatants was profiled at 48 h after the first illumination. All data represent the mean ± SD; n = 3 independent experiments. The orange frame marks the highest-fold induction mediated by the FISC system. Source data is available in the Source Data file.

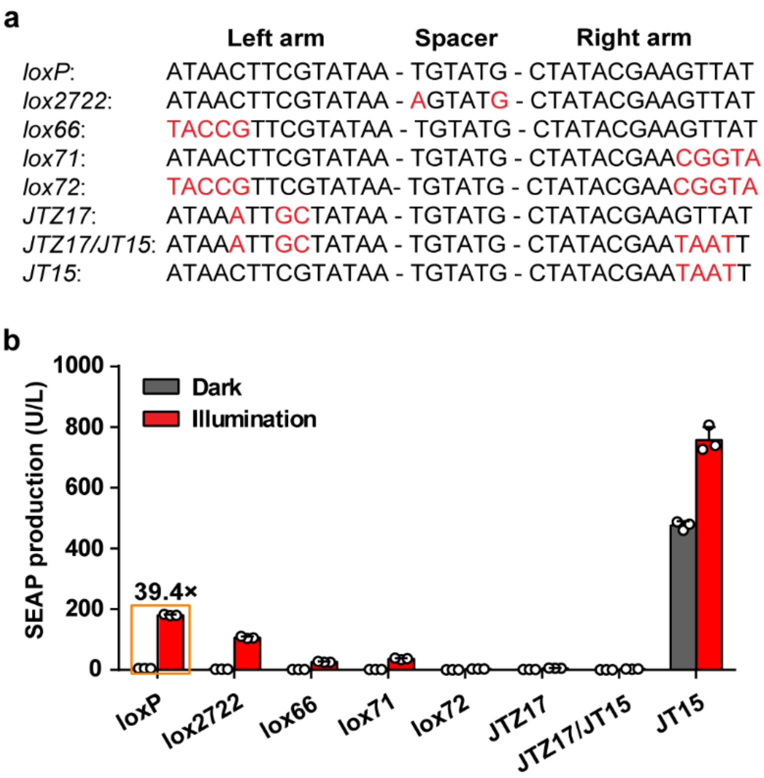

Supplementary Figure 4. The catalytic activity of CreN59/CreC60 for different *loxP* mutants. (**a**) Different mutated *loxP* sequences and mutations are highlighted in red. (**b**) Optimization the CreN59/CreC60 catalytic activity for different *loxP* mutants. 6×10^4 HEK-293 cells were co-transfected with pXY137 (100 ng), pXY169 (10 ng), pXY177 (10 ng) and different plasmids encoding *loxP* mutants (pGY125/pXY229/pXY230/pXY231/pXY232/pXY234/pXY235/pXY233, 200 ng), and then illuminated for 6 h with FRL (1.5 mW cm^{-2}, 730 nm) each day for two days. SEAP expression in the culture supernatants was profiled at 48 h after the first illumination. Data represent the mean ± SD; *n* = 3 independent experiments. The orange frame marks the highest-fold induction mediated by the FISC system in this experiment. Source data is available in the Source Data file.

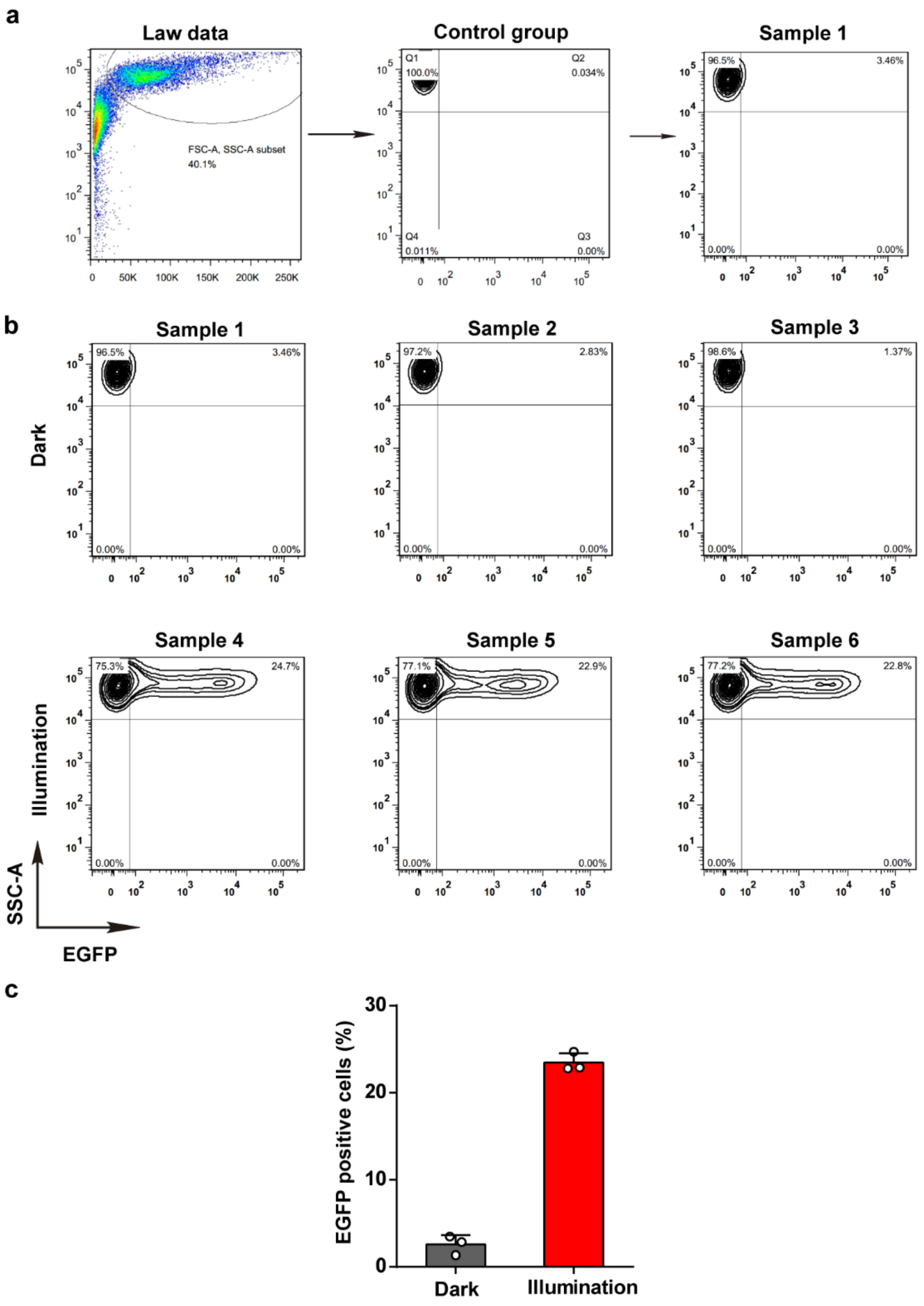

Supplementary Figure 5. Flow cytometric analysis of FISC-mediated DNA recombination efficiency. (a) Gating strategy to determine the percentage of Cre-catalyzed DNA recombination mediated by the FISC system. (**b**) Flow cytometry contour plots showing Cre-catalyzed DNA recombination mediated by FISC system. HEK-293 cells (6 × 10^4) were co-transfected with pXY137, pXY169 (P_{hCMV}-CreN59-L9-Coh2-NES-pA), pXY177 (P_{FRLd}-NLS-DocS-L9-CreC60-pA), and pDL78 (P_{hCMV}-*loxP*-STOP-*loxP*-EGFP-pA) at a ratio of 10:1:1:20 (w/w/w/w), illuminated for 6 h with FRL (1.5 mW cm^{-2}, 730 nm) each day for two days. The expression of the reporter

EGFP was determined by flow cytometry at 48 h after the first illumination. **(c)** Quantification of the percentage of HEK-293 cells expressing EGFP shown in **b**. Data represent the mean ± SD; $n = 3$ independent experiments. Source data is available in the Source Data file.

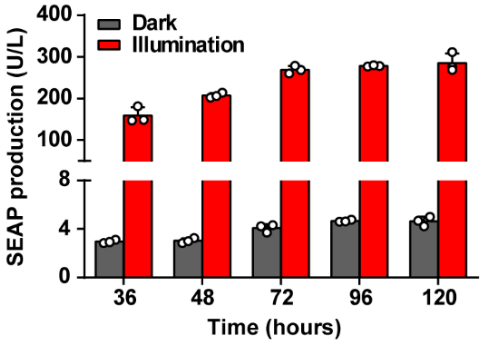

Supplementary Figure 6. Time-dependent SEAP production mediated by the FISC system. 6 × 10^4 HEK-293 cells were co-transfected with pXY137, pXY169, pXY177 and pGY125 (SEAP reporter) at a 10:1:1:20 (w/w/w/w) ratio and illuminated with FRL (1.5 mW cm^{-2}, 730 nm) for 6 h each day for two days. SEAP levels were profiled at different time points after the first illumination. All data represent the mean ± SD; $n = 3$ independent experiments. Source data is available in the Source Data file.

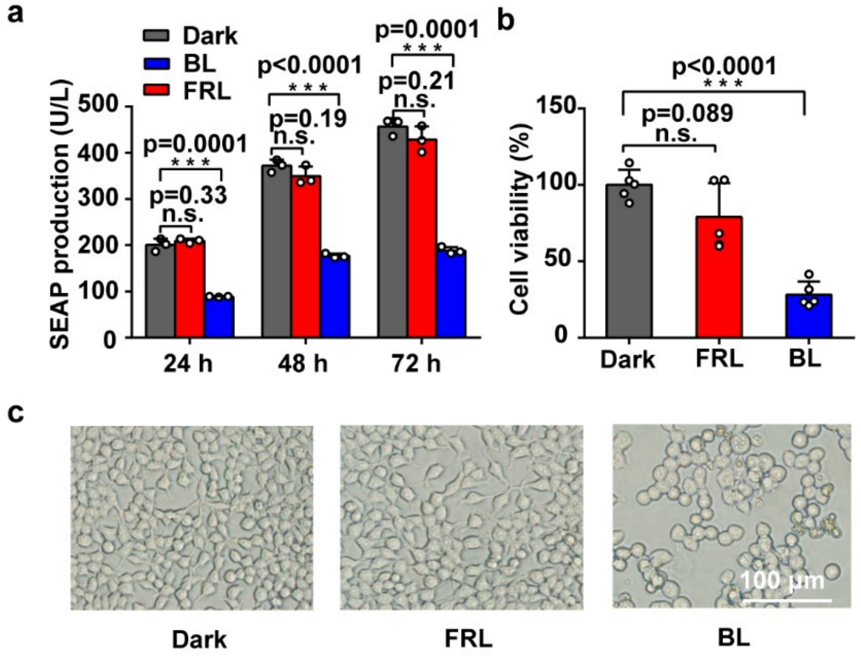

Supplementary Figure 7. Comparison of the phototoxicity of FRL and blue light (BL) on mammalian cells. (**a**) Protein-based metabolic integrity assay of human cells

exposed to FRL and BL. 6 × 10^4 HEK-293 cells seeded in a 24-well plate were transfected with pSEAP2-control (P_{SV40}-SEAP-pA, 50 ng) and illuminated for 24, 48, 72 h (1.5 mW cm^{-2}, 730 nm or 460 nm), SEAP expression levels in culture supernatants were profiled. Data represent the mean ± SD; n = 3 independent experiments. Comparisons were made with Two-tailed t test: ***P<0.001, Dark v.s. BL; n.s., not significant, Dark v.s. FRL. (**b**) Cell viability of human cells after exposure to FRL or BL, respectively. 1 × 10^4 HEK-293 cells seeded in a 96-well plate were illuminated with FRL (1.5 mW cm^{-2}, 730 nm) or BL (1.5 mW cm^{-2}, 460 nm) for 24 h and cell viability was assayed using Cell Counting Kit-8. Data represent the mean ± SD; n = 5 independent experiments. Comparisons were made with Two-tailed t test: ***P<0.001, Dark v.s. BL; n.s., not significant, Dark v.s. FRL. (**c**) A representative of bright field microscope image of HEK-293 cells exposed to FRL or BL. Control cells were kept in the dark. Representative images from n = 2 biological replicates. Scale bar, 100 μm. Source data is available in the Source Data file.

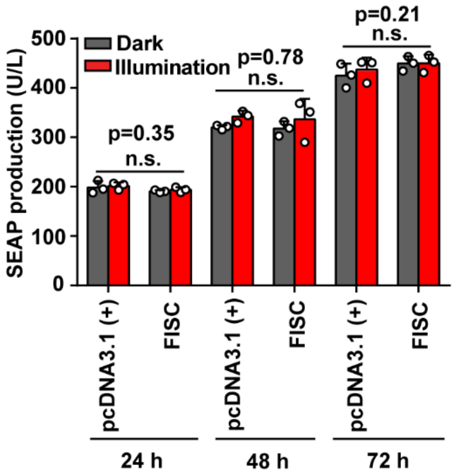

Supplementary Figure 8. Impact of ectopic FISC constituents' expression and phototoxicity on human cells. 6 × 10^4 HEK-293 cells were co-transfected with pSEAP2-control (50 ng), pXY137 (100 ng), pXY169 (10 ng), pXY177 (10 ng), and illuminated with FRL (1.5 mW cm^{-2}, 730 nm) for 6 h every 24 h and daily SEAP expression were compared against control cells co-transfected with pSEAP2-control and pcDNA3.1(+) (50 ng/120 ng). Data represent the mean ± SD (n = 3 independent experiments). Comparisons were made with Two-tailed t test: n.s., not significant, FISC v.s. pcDNA3.1. Source data is available in the Source Data file.

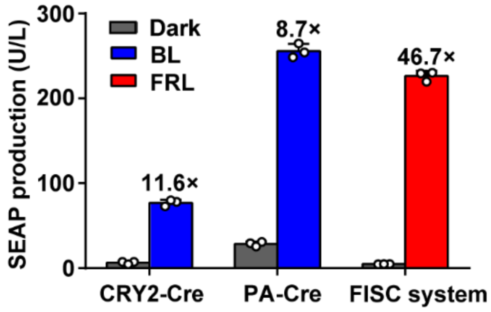

Supplementary Figure 9. Comparison of the FISC system with CRY2-Cre and PA-Cre system *in vitro*. 6 × 10^4 HEK-293 cells seeded in a 24-well plate were co-transfected with FISC system [pXY137 (100 ng)/pXY169 (10 ng)/pXY177 (10 ng)/pGY125 (200 ng)] or the CRY2-Cre [pXY240 (14.8 ng)/pGY125 (200 ng)/pcDNA3.1(+) (105.2 ng)] or PA-Cre [PA-Cre (13.4 ng)/pGY125 (200 ng)/pcDNA3.1(+) (106.6 ng)], and then illuminated for 6 h with FRL (1.5 mW cm^{-2}, 730 nm) or BL (1.5 mW cm^{-2}, 460 nm) each day for two days. SEAP expression in the culture supernatants was profiled at 48 h after the first illumination. Data represent the mean ± SD; $n = 3$ independent experiments. Source data is available in the Source Data file.

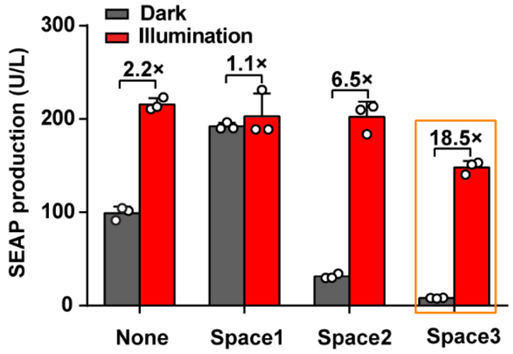

Supplementary Figure 10. Optimization of the different distance between promoters for two split-Cre sequences in a single plasmid. 6 × 10^4 HEK-293 cells seeded in a 24-well plate were co-transfected with pXY137 (150 ng), pGY125 (200 ng) and the different plasmid encoding two split-Cre fusion fragments pXY202 (pA-CreC60-L9-DocS-NLS-P_{FRLd}::P_{hCMV}-CreN59-L9-Coh2-NES-pA, 100 ng; no space), or pXY203 (pA-CreC60-L9-DocS-NLS-P_{FRLd}-Space1-P_{hCMV}-CreN59-L9-Coh2-NES-P2A-ZeoR-pA, 100 ng; Space1, ~1500 bp), or pXY204 (pA-CreC60-L9-DocS-NLS-P_{FRLd}-Space2-P_{hCMV}-CreN59-L9-Coh2-NES-P2A-ZeoR-pA, 100 ng; Space2, ~3000 bp) or pXY237 (pA-CreC60-L9-DocS-NLS-P_{FRLd}-Space3-P_{hCMV}-CreN59-L9-Coh2-NES-P2A-ZeoR-pA, 100 ng; Space3, ~4000 bp) and then illuminated for 6 h with FRL (1.5

mW cm^{-2}, 730 nm) each day for two days. SEAP expression in the culture supernatants was profiled at 48 h after the first illumination. All data represent the mean ± SD; n = 3 independent experiments. The orange frame marks the highest-fold induction mediated by the FISC system in this experiment. Source data is available in the Source Data file.

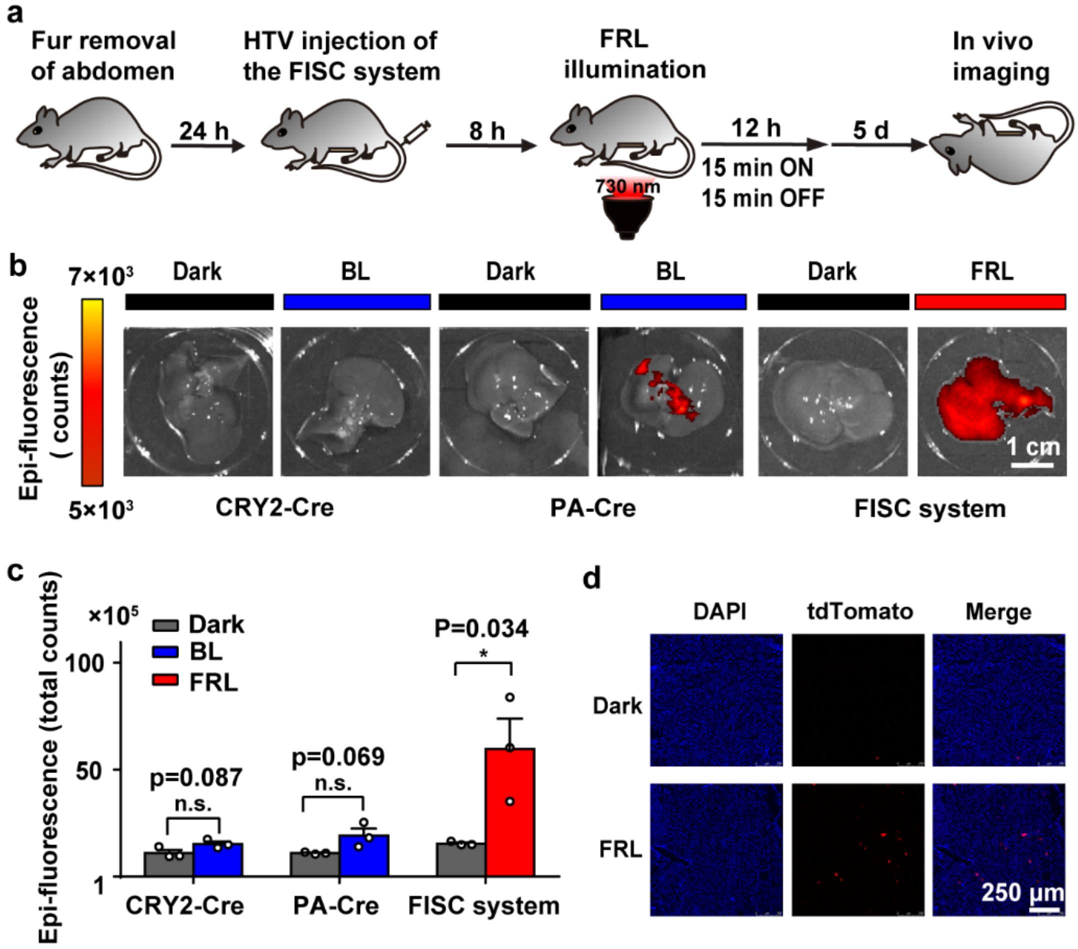

Supplementary Figure 11. FISC-mediated DNA recombination in transgenic Cre-tdTomato reporter mice via hydrodynamic injection. (**a**) Schematic showing the experimental procedure for light-induced DNA recombination activity in the mouse liver. (**b**) Comparison of the tdTomato expression in the isolated liver tissue of mice injected with the FISC system or with the CRY2-Cre or PA-Cre systems. The mice were hydrodynamically injected with a FISC system iteration comprising two plasmids [pXY137 (150 µg), pXY237 (100 µg)] or the CRY2-Cre system (67 µg) or the PA-Cre system (74 µg) with the same molar concentration of Cre. Eight hours after injection, the mice were kept in the dark or illuminated with FRL (20 mW cm^{-2}, 730 nm) or BL (20 mW cm^{-2}, 460 nm) for 12 h (15 min on, 15 min off, alternating). Scale bar, 1 cm.

(**c**) Quantification of the tdTomato expression shown in **b**. Data represent the mean ± SEM (n = 3 mice/group). Comparisons were made with Two-tailed t test: n.s., not significant, Dark v.s. BL; *P < 0.05 Dark v.s. FRL. (**d**) Representative fluorescence images of the mouse liver sections shown in **b** (n = 5 biological replicates. Scale bar, 250 μm). Source data is available in the Source Data file.

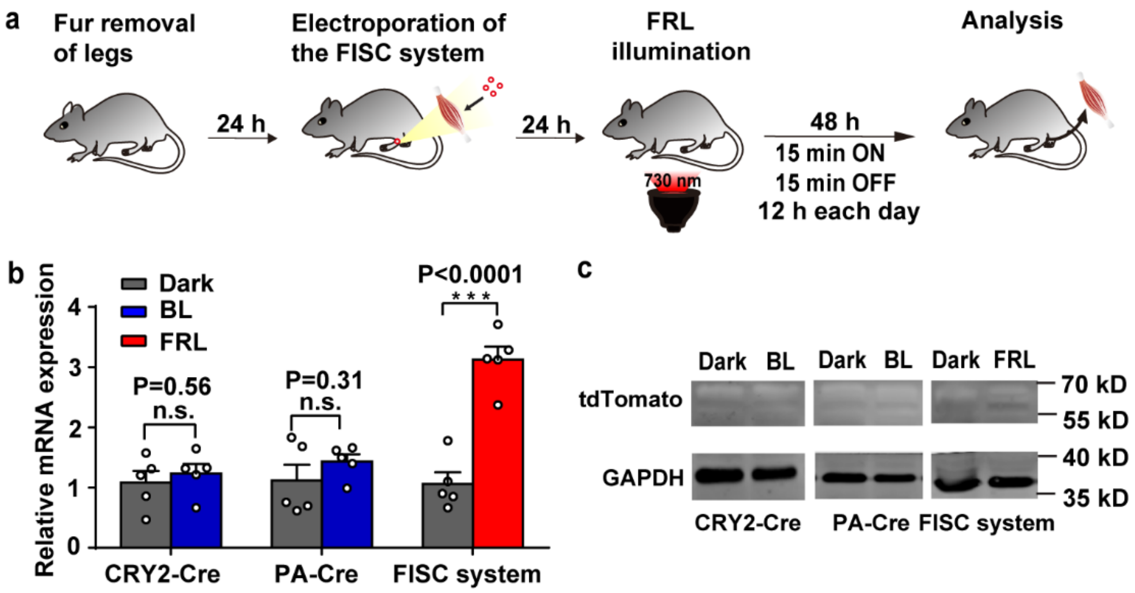

Supplementary Figure 12. FISC-mediated DNA recombination in the mussel of transgenic Cre-tdTomato reporter mice using electroporation. (**a**) Schematic showing the experimental procedure for light-induced DNA recombination activity in mouse muscle. qRT-PCR (**b**) and Western blotting (**c**) analysis of tdTomato expression in Cre-tdTomato reporter mice. The light-inducible system components—CRY2-Cre system (40 μg), the PA-Cre system (40 μg), or a FISC system iteration comprising the pXY137 (20 μg) and pXY237 (20 μg) plasmids were delivered into the tibialis posterior muscles of the mice. The electroporated mice were kept in the dark or illuminated with FRL (20 mW cm^{-2}, 730 nm) or BL (20 mW cm^{-2}, 460 nm) for 12 h (15 min on, 15 min off) each day for two days. Muscles were collected at 3 days after electroporation, and tdTomato expression was measured by qRT-PCR (**b**) and Western blotting (**c**). Data in **b** represent the mean ± SEM (n = 5 mice/group). Comparisons were made with Two-tailed t test: n.s., not significant, Dark v.s. BL; ***P < 0.001 Dark v.s. FRL.Western blotting images are representative of three mice from two independent experiments. Source data is available in the Source Data file.

Supplementary Table 1. Different linker amino acid sequences

name	amino acid sequence	linker
L1	GT	short linker
L2	LEASTGGSGT	flexible linker
L3	ASPSNPGASNGS	semi-flexible linker
L4	LEASPSNPGASNGSGT	semi-flexible linker
L5	DD	short linker
L6	DV	short linker
L7	EQ	short linker
L8	GGGDV	flexible linker
L9	LEASPSNPGASNGS	semi-flexible linker
L10	LEASPSNPGASN	semi-flexible linker
L11	LEASPSNPGA	semi-flexible linker
L12	GGGGSGGGGSGGGGR	flexible linker

Supplementary Table 2. Different combinations of the linkers in Docs-CreC60 or Coh2-CreN59

Docs-Linker-CreC60 \ CreN59-Linker-Coh2	pXY157 (L1)	pXY158 (L2)	pXY159 (L3)	pXY160 (L4)	pXY173 (L5)	pXY174 (L6)	pXY175 (L7)	pXY176 (L8)	pXY177 (L9)	pXY178 (L10)	pXY179 (L11)
pXY146 (L1)	L1/L1	L1/L2	L1/L3	L1/L4	-	-	-	-	-	-	-
pXY147 (L2)	L2/L1	L2/L2	L2/L3	L2/L4	-	-	-	-	-	-	-
pXY150 (L3)	L3/L1	L3/L2	L3/L3	L3/L4	-	-	-	-	-	-	-
pXY151 (L4)	L4/L1	L4/L2	L4/L3	L4/L4	-	-	-	-	-	-	-
pXY165 (L5)	-	-	-	-	L5/L5	L5/L6	L5/L7	L5/L8	L5/L9	L5/L10	L5/L11
pXY166 (L6)	-	-	-	-	L6/L5	L6/L6	L6/L7	L6/L8	L6/L9	L6/L10	L6/L11
pXY167 (L7)	-	-	-	-	L7/L5	L7/L6	L7/L7	L7/L8	L7/L9	L7/L10	L7/L11
pXY168 (L8)	-	-	-	-	L8/L5	L8/L6	L8/L7	L8/L8	L8/L9	L8/L10	L8/L11
pXY169 (L9)	-	-	-	-	L9/L5	L9/L6	L9/L7	L9/L8	L9/L9	L9/L10	L9/L11
pXY170 (L10)	-	-	-	-	L10/L5	L10/L6	L10/L7	L10/L8	L10/L9	L10/L10	L10/L11
pXY171 (L11)	-	-	-	-	L11/L5	L11/L6	L11/L7	L11/L8	L11/L9	L11/L10	L11/L11

Supplementary Table 3. Comparison of the FISC-mediated DNA recombination efficiency using three different delivery methods in transgenic Cre-tdTomato reporter mice

<table>
<tr><th>Delivery methods</th><th>Bioluminescence measurements of isolated liver tissue</th><th>qPCR analysis</th><th>Western blot analysis</th><th>Fluorescence images of liver sections</th></tr>
<tr><td>Hydrodynamic injection</td><td>3.5-fold</td><td>/</td><td>/</td><td>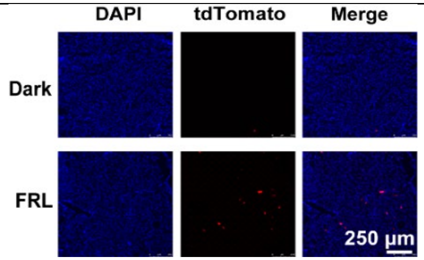
</td></tr>
<tr><td>Electroporation</td><td>/</td><td>2.9-fold</td><td>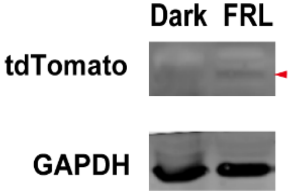
</td><td>/</td></tr>
<tr><td>AAV transduction</td><td>20.4-fold</td><td>4.7-fold</td><td>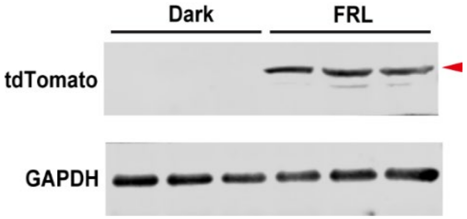
</td><td>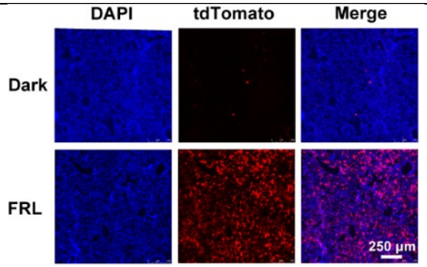
</td></tr>
</table>

Data were expressed as fold change of FISC-mediated DNA recombination in FRL-illuminated mice compared with control mice kept in the dark.

Supplementary Table 4. Plasmids designed and used in this study

Plasmid	Description and Cloning Strategy	Reference
pcDNA3.1(+)	Constitutive P_{hCMV}-driven mammalian expression vector [P_{hCMV}-MCS-pA].	Invitrogen' CA
pSEAP2-control	Constitutive P_{SV40}-driven SEAP expression vector [P_{SV40}-SEAP-pA].	Clontech' CA
PA-Cre	Constitutive P_{hCMV}-driven CreN59-nMag and pMag-CreC60 expression vector [P_{hCMV}-CreN59-L1-nMag-NLS-P2A-NLS-pMag-L1-CreC60-pA].	Kawano, *et al.*[1]
pXY34	FRTA-specific FRL-inducible SEAP expression vector [P_{FRLa}-SEAP-pA; P_{FRLa}, pA-(whiG)$_3$-$P_{hCMVmin}$].	J. Shao, *et al.*[2]
pXY110	Constitutive P_{hCMV}-driven CreN59-L0-Coh2 expression vector [P_{hCMV}-CreN59-L0-Coh2-NES-pA].	This work
pXY111	FRTA-specific FRL-inducible DocS-L0-CreC60 expression vector [P_{FRLa}-NLS-DocS-L0-CreC60-pA].	This work
pXY121	FRTA-specific FRL-inducible DocS-L0-CreC60 expression vector [P_{FRLc}-NLS-DocS-L0-CreC60-pA; P_{FRLc}, pA-(whiG)$_3$-P_{min}].	This work
pXY133	FRTA-specific FRL-inducible DocS-L0-CreC60 expression vector [P_{FRLd}-NLS-DocS-L0-CreC60-pA; P_{FRLd}, pA-(whiG)$_3$-TATA].	This work
pXY137	Constitutive P_{hCMV}-driven BldD, BphS and YhjH expression vector [P_{hCMV}-p65-VP64-L0-BldD-pA::P_{hCMV} -BphS-P2A-YhjH-pA].	This work
pXY146	Constitutive P_{hCMV}-driven CreN59-L1-Coh2 expression vector [P_{hCMV}-CreN59-L1-Coh2-NES-pA].	This work
pXY147	Constitutive P_{hCMV}-driven CreN59-L2-Coh2 expression vector [P_{hCMV}-CreN59-L2-Coh2-NES-pA].	This work
pXY150	Constitutive P_{hCMV}-driven CreN59-L3-Coh2 expression vector [P_{hCMV}-CreN59-L3-Coh2-NES-pA].	This work
pXY151	Constitutive P_{hCMV}-driven CreN59-L4-Coh2 expression vector [P_{hCMV}-CreN59-L4-Coh2-NES-pA].	This work
pXY157	FRTA-specific FRL-inducible DocS-L1-CreC60 expression vector [P_{FRLd}-NLS-DocS-L1-CreC60-pA].	This work
pXY158	FRTA-specific FRL-inducible DocS-L2-CreC60 expression vector [P_{FRLd}-NLS-DocS-L2-CreC60-pA].	This work
pXY159	FRTA-specific FRL-inducible DocS-L3-CreC60 expression vector [P_{FRLd}-NLS-DocS-L3-CreC60-pA].	This work
pXY160	FRTA-specific FRL-inducible DocS-L4-CreC60 expression vector [P_{FRLd}-NLS-DocS-L4-CreC60-pA].	This work
pXY165	Constitutive P_{hCMV}-driven CreN59-L5-Coh2 expression vector [P_{hCMV}-CreN59-L5-Coh2-NES-pA].	This work

pXY166	Constitutive P_{hCMV}-driven CreN59-L6-Coh2 expression vector [P_{hCMV}-CreN59-L6-Coh2-NES-pA].	This work
pXY167	Constitutive P_{hCMV}-driven CreN59-L7-Coh2 expression vector [P_{hCMV}-CreN59-L7-Coh2-NES-pA].	This work
pXY168	Constitutive P_{hCMV}-driven CreN59-L8-Coh2 expression vector [P_{hCMV}-CreN59-L8-Coh2-NES-pA].	This work
pXY169	Constitutive P_{hCMV}-driven CreN59-L9-Coh2 expression vector [P_{hCMV}-CreN59-L9-Coh2-NES-pA].	This work
pXY170	Constitutive P_{hCMV}-driven CreN59-L10-Coh2 expression vector [P_{hCMV}-CreN59-L10-Coh2-NES-pA].	This work
pXY171	Constitutive P_{hCMV}-driven CreN59-L11-Coh2 expression vector [P_{hCMV}-CreN59-L11-Coh2-NES-pA].	This work
pXY173	FRTA-specific FRL-inducible DocS-L5-CreC60 expression vector [P_{FRLd}-NLS-DocS-L5-CreC60-pA].	This work
pXY174	FRTA-specific FRL-inducible DocS-L6-CreC60 expression vector [P_{FRLd}-NLS-DocS-L6-CreC60-pA].	This work
pXY175	FRTA-specific FRL-inducible DocS-L7-CreC60 expression vector [P_{FRLd}-NLS-DocS-L7-CreC60-pA].	This work
pXY176	FRTA-specific FRL-inducible DocS-L8-CreC60 expression vector [P_{FRLd}-NLS-DocS-L8-CreC60-pA].	This work
pXY177	FRTA-specific FRL-inducible DocS-L9-CreC60 expression vector [P_{FRLd}-NLS-DocS-L9-CreC60-pA].	This work
pXY178	FRTA-specific FRL-inducible DocS-L10-CreC60 expression vector [P_{FRLd}-NLS-DocS-L10-CreC60-pA].	This work
pXY179	FRTA-specific FRL-inducible DocS-L11-CreC60 expression vector [P_{FRLd}-NLS-DocS-L11-CreC60-pA].	This work
pXY185	Cre-inducible and P_{hCMV}-driven luciferase expression vector [P_{hCMV}-*loxP*-STOP-*loxP*-Luciferase-pA].	This work
pXY 202	FRTA-specific FRL-inducible Docs-L9-CreC60 expression and constitutive P_{hCMV}-driven CreN59-L9-Coh2 expression vector [pA-CreC60-L9-DocS-NLS-P_{FRLd}::P_{hCMV}-CreN59-L9-Coh2-NES-pA].	This work
pXY203	FRTA-specific FRL-inducible Docs-L9-CreC60 expression and constitutive P_{hCMV}-driven CreN59-L9-Coh2 expression vector [pA-CreC60-L9-DocS-NLS-P_{FRLd}-Space1-P_{hCMV}-CreN59-L9-Coh2-NES-P2A-ZeoR-pA].	This work
pXY204	FRTA-specific FRL-inducible Docs-L9-CreC60 expression and constitutive P_{hCMV}-driven CreN59-L9-Coh2 expression vector [pA-CreC60-L9-DocS-NLS-P_{FRLd}-Space2-P_{hCMV}-CreN59-L9-Coh2-NES-P2A-ZeoR-pA].	This work
pXY229	Cre-inducible and P_{hCMV}-driven SEAP expression vector [P_{hCMV}-*lox2272*-STOP-*lox2272*-SEAP-pA].	This work
pXY230	Cre-inducible and P_{hCMV}-driven SEAP expression vector [P_{hCMV}-*lox66*-STOP-*lox66*-SEAP-pA].	This work
pXY231	Cre-inducible and P_{hCMV}-driven SEAP expression vector [P_{hCMV}-*lox71*-STOP-*lox71*-SEAP-pA].	This work
pXY232	Cre-inducible and P_{hCMV}-driven SEAP expression vector [P_{hCMV}-*lox72*-STOP-*lox72*-SEAP-pA].	This work
pXY233	Cre-inducible and P_{hCMV}-driven SEAP expression vector [P_{hCMV}-*JT15*-STOP-*JT15*-SEAP-pA].	This work
pXY234	Cre-inducible and P_{hCMV}-driven SEAP expression vector [P_{hCMV}-*JTZ17*-STOP-*JTZ17*-SEAP-pA].	This work
pXY235	Cre-inducible and P_{hCMV}-driven SEAP expression vector [P_{hCMV}-*JT15-JTZ17*-STOP-*JT15-JTZ17*-SEAP-pA].	This work
pXY237	FRTA-specific FRL-inducible CreC60-L9-DocS expression and constitutive P_{hCMV}-driven CreN59-L9-Coh2 expression vector [pA-CreC60-L9-DocS-NLS-P_{FRLd}-Space3-P_{hCMV}-CreN59-L9-Coh2-NES-P2A-ZeoR-pA].	This work
pXY240	Constitutive P_{hCMV}-driven CIB1-L12-CreC106 and CRY2-L12-CreN104 expression vector [P_{hCMV}-CIB1-L12-	Taslimi, A.

	CreC106-IRES-CRY2-L12-CreN104-pA].	*et al.*[3]
pCRE5	AAV vector encoding constitutive P_{hCMV}-driven expression unit for CreN59-L9-Coh2 [ITR-P_{hCMV}-CreN59-L9-Coh2-NES-pA-ITR].	This work
pCRE14	AAV vector encoding constitutive P_{hCMV}-driven expression unit for BphS [ITR-P_{hCMV}-BphS-pA-ITR].	This work
pCRE23	AAV vector encoding constitutive P_{hCMV}-driven expression unit for BldD and FRTA-specific FRL-inducible expression unit for Docs-L9-CreC60 [ITR-P_{hCMV}-p65-VP64-BldD-pA::P_{FRLd}-NLS-DocS-L9-CreC60-pA-ITR].	This work
pGY 125	Cre-inducible and P_{hCMV}-driven SEAP expression vector [P_{hCMV}-*loxP*-STOP-*loxP*-SEAP-pA].	This work
pDL78	Cre-inducible and P_{hCMV}-driven EGFP expression vector [P_{hCMV}-*loxP*-STOP-*loxP*-EGFP-pA].	This work

Abbreviations: **BldD**, Streptomyces coelicolor transcription factor regulating hyphae formation; **BphS**, engineered bacterial diguanylate cyclase; **CIB1**, Cryptochrome-interacting basic-helix-loop-helix 1; **Coh2**, anchoring proteins of *C. thermocellum*; **Cre**, Cyclization Recombination Enzyme; **CRY2**, Cryptochrome 2 in Arabidopsis thaliana; **DocS**, *C. thermocellum* interacting partner of Coh2; **EGFP**, enhanced green fluorescent protein; **FRL**, far-red light; **FRTA**, mammalian far-red light-dependent transactivator (p65-VP64-BldD); **FISC**, far-red light-induced split Cre-*loxP* system; **GOI**, gene of interest; **IRES**, internal ribosome entry site; **ITR,** inverted terminal repeat; ***loxP***, a 34 bp sequence from P1 phage that Cre recombinase binds (5'-ATAACTTCGTATAGCATACATTATACGAAGTTAT-3'); **NES**, nuclear export signal; **NLS,** nuclear localization signal; **P2A**, picornavirus-derived self-cleaving peptide engineered for bicistronic gene expression in mammalian cells; **p65**, 65 kDa transactivator subunit of NF-κB; **pA**, polyadenylation signal; **PCR**, polymerase chain reaction; **P_{FRLx}**, BldD-based synthetic mammalian far-red light-inducible promoter variants; **P_{hCMV}**, human cytomegalovirus immediate early promoter; **$P_{hCMVmin}$**, minimal version of P_{hCMV}; **P_{min}**, minimal eukaryotic promoter; **ROI**, region of interest; **SEAP**, human placental secreted alkaline phosphatase; **STOP**, a long fragment contains polyadenylation signal to prevent transcription; **TATA,** minimal eukaryotic promoter with only TATA box; **VP64**, tetrameric core of Herpes simplex virus-derived transactivation domain; **whiG**, BldD-specific binding sequence; **YhjH**, bacterial c-di-GMP phosphodiester.

Supplementary Table 5. Primers used in this study

name	Forward Primers (5' to 3')	Reverse Primer (5' to 3')
CreN59	ATGTCCAATTTACTGACCG	ATTCAACTTGCACCATGCCG
Coh2-NES	GTGGTGGTGGAGATCGGCAAGG	CAGCTGGTAGGCGTACAGGATC
NLS-DocS	GCCAGTCCCAAGAAGAAGAGAAAGGTGGAGGC<br>GTAGCACCAAGCTGTACGGCGAC	GTTCTTGTAGGGCAGGGTGTC
CreC60	AACCGGAAATGGTTTCCCGC	ATCCCCATCTTCCAGCAGGC
Pmin	ggGTCGACAGCGGAGACTCTAGAGGGTATATAAT<br>GGAAGCTCGACTTCCAGCTTGGCAATCCGGTAC<br>TGTTGGTAAAg	aattcTTTACCAACAGTACCGGATTGCCAAGCTGGAA<br>GTCGAGCTTCCATTATATACCCTCTAGAGTCTCCGC<br>TGTCGACcctgca
TATA	ggAGAGGGTATATAATGGAAGCTCGAATTCCAGA<br>AGCTTATACTCAGTGCCCTGACTATATACTCAGT<br>GCCCTGACTATg	aattcATAGTCAGGGCACTGAGTATATAGTCAGGGCAC<br>TGAGTATAAGCTTCTGGAATTCGAGCTTCCATTATAT<br>ACCCTCTcctgca
Luciferase	GCCACCATGGAAGACGCCAA	CTCGAAGCGGCCGGCCGCCCCGAC
lox2272-STOP-*lox2272*	ATAACTTCGTATAAAGTATCCTATACGAAGTTATG<br>TCCGAACAAACGACCCAACACCCG	ATAACTTCGTATAGGATACTTTATACGAAGTTATACT<br>TACCATGTCAGATCCAGACATGATA
lox66-STOP-*lox66*	TACCGTTCGTATAATGTATGCTATACGAAGTTATG<br>GATCCGAACAAACGACCCAACACCCG	ATAACTTCGTATAGCATACATTATACGAACGGTAACT<br>TACCATGTCAGATCCAGACATGATA
lox71-STOP-*lox71*	ATAACTTCGTATAATGTATGCTATACGAACGGTAG	TACCGTTCGTATAGCATACATTATACGAAGTTATACT

	ATCCGAACAAACGACCCAACACCCG	TACCATGTCAGATCCAGACATGAT
lox72-STOP-*lox72*	TACCGTTCGTATAATGTATGCTATACGAACGGTA GGATCCGAACAAACGACCCAACACCCG	TACCGTTCGTATAGCATACATTATACGAACGGTAACT TACCATGTCAGATCCAGACATGAT
JT15-STOP-*JT15*	ATAACTTCGTATAATGTATGCTATACGAATAATTG GATCCGAACAAACGACCCAACACCCG	AATTATTCGTATAGCATACATTATACGAAGTTATACTT ACCATGTCAGATCCAGACATGAT
JTZ17-STOP-*JTZ17*	ATAAATTGCTATAATGTATGCTATACGAAGTTATG GATCCGAACAAACGACCCAACACCCG	ATAACTTCGTATAGCATACATTATAGCAATTTATACTT ACCATGTCAGATCCAGACATGAT
JT15-*JTZ17*-STOP-*JT15*-*JTZ17*	ATAAATTGCTATAATGTATGCTATACGAATAATTG GATCCGAACAAACGACCCAACACCCG	AATTATTCGTATAGCATACATTATAGCAATTTATACTT ACCATGTCAGATCCAGACATGAT
BphS	GATGTACGGGCCAGATATACGC	GGTTCTTTCCGCCTCAGAAG

Amino acids or DNA sequence information

1. FRTA: p65-VP64-Linker-BldD

MPSGQISNQALALAPSSAPVLAQTMVPSSAMVPLAQPPAPAPVLTPGPPQSLMGSGRADA
LDDFDLDMLGSDALDDFDLDMLGSDALDDFDLDMLGSDALDDFDLDMLINASGSGGGG
DVMASPKKKRKVEASSSEYAKQLGAKLRAIRTQQGLSLHGVEEKSQGRWKAVVVGSYER
GDRAVTVQRLAELADFYGVPVQELLPGTTPGGAAEPPPKLVLDLERLAHVPQEKAGPLQR
YAATIQSQRGDYNGKVLSIRQDDLRTLAVIYDQSPSVLTEQLISWGVLDADARRAVAHEEN

2. FRS: BphS-P2A-YhjH

MARGCLMTISGGTFDPSICEMEPIATPGAIQPHGALMTARADSGRVAHASVNLGEILGLPA
ASVLGAPIGEVIGRVNEILLREARRSGSETPETIGSFRRSDGQLLHLHAFQSGDYMCLDIEP
VRDEDGRLPPGARQSVIETFSSAMTQVELCELAVHGLQLVLGYDRVMAYRFGADGHGEVI
AERRRQDLEPYLGLHYPASDIPQIARALYLRQRVGAIADACYRPVPLLGHPELDDGKPLDL
THSSLRSVSPVHLDYMQNMNTAASLTIGLADGDRLWGMLVCHNTTPRIAGPEWRAAAG
MIGQVVSLLLSRLGEVENAAETLARQSTLSTLVERLSTGDTLAAAFVAADQLILDLVGASA
AVVRLAGQELHFGRTPPVDAMQKVLDSLGRPSPLEVLSLDDVTLRHPELPELLAAGSGILL
LPLTSGDGDLIAWFRPEHVQTITWGGNPAEHGTWNPATQRMRPRASFDAWKETVTGRSLP
WTSAERNCARELGEAIAAEMAQRTRAEELERVAMVDSLTRLWNRLGIETLLKREWEYATR
KNSPISIVMIDFDNFKQINDQHGHLVGDEVLQGSARLIISVLASYDILGRWGGDEFMLILPG
SGREQTAVLLERIQATIAQNPVPTSAGPMAISLSMGGVSVFTNQGEALQYWVEQADNQLM
KVKRLGKGNFQLAEYHHHHHHGSGATNFSLLKQAGDVEENPGPSGIRQVIQRISNPEASIE
SLQERRFWLQCERAYTWQPIYQTCGRLMAVELLTVVTHPLNPSQRLPPDRYFTEITVSHR
MEVVKEQIDLLAQKADFFIEHGLLASVNIDGPTLIALRQQPKILRQIERLPWLRFELVEHIR
LPKDSTFASMCEFGPLWLDDFGTGMANFSALSEVRYDYIKIARELFVMLRQSPEGRTLFSQ
LLHLMNRYCRGVIVEGVETPEEWRDVQNSPAFAAQGWFLSRPAPIETLNTAVLAL

3. Cre

MSNLLTVHQNLPALPVDATSDEVRKNLMDMFRDRQAFSEHTWKMLLSVCRSWAAWCKL
NNRKWFPAEPEDVRDYLLYLQARGLAVKTIQQHLGQLNMLHRRSGLPRPSDSNAVSLVM
RRIRKENVDAGERAKQALAFERTDFDQVRSLMENSDRCQDIRNLAFLGIAYNTLLRIAEIA
RIRVKDISRTDGGRMLIHIGRTKTLVSTAGVEKALSLGVTKLVERWISVSGVADDPNNYLFC
RVRKNGVAAPSATSQLSTRALEGIFEATHRLIYGAKDDSGQRYLAWSGHSARVGAARDMA
RAGVSIPEIMQAGGWTNVNIVMNYIRNLDSETGAMVRLLEDGD

4. P_{FRLa}: pA-3×whiG-$P_{hCMVmin}$

CAGACATGATAAGATACATTGATGAGTTTGGACAAACCACAACTAGAATGCAGTGAAA
AAAATGCTTTATTTGTGAAATTTGTGATGCTATTGCTTTATTTGTAACCATTATAAGCTGC
AATAAACAAGTTAACAACAACAATTGCATTCATTTTATGTTTCAGGTTCAGGGGGAGGT

GTGGGAGGTTTTTTAAACCTCACGCTACGCTCACTCACGCTACGCTCACTCACGCTACG
CTCACCTGCAGGTCGAGCTCGGTACCCGGGTCGAGTAGGCGTGTACGGTGGGAGGCCT
ATATAAGCAGAGCTCGTTTAGTGAACCGTCAGATCGCCTGGAGACGCCATCCACGCTG
TTTTGACCTCCATAGAAGACACCGGGACCGATCCAGCCTCCGCG

5. **P_{FRLc}: pA-3×whiG-P_{min}**
CAGACATGATAAGATACATTGATGAGTTTGGACAAACCACAACTAGAATGCAGTGAAA
AAAATGCTTTATTTGTGAAATTTGTGATGCTATTGCTTTATTTGTAACCATTATAAGCTGC
AATAAACAAGTTAACAACAACAATTGCATTCATTTTATGTTTCAGGTTCAGGGGGAGGT
GTGGGAGGTTTTTTAAACCTCACGCTACGCTCACTCACGCTACGCTCACTCACGCTACG
CTCACCTGCAGGGTCGACAGCGGAGACTCTAGAGGGTATATAATGGAAGCTCGACTTC
CAGCTTGGCAATCCGGTACTGTTGGTAAA

6. **P_{FRLd}: pA-3×whiG-TATA**
CAGACATGATAAGATACATTGATGAGTTTGGACAAACCACAACTAGAATGCAGTGAAA
AAAATGCTTTATTTGTGAAATTTGTGATGCTATTGCTTTATTTGTAACCATTATAAGCTGC
AATAAACAAGTTAACAACAACAATTGCATTCATTTTATGTTTCAGGTTCAGGGGGAGGT
GTGGGAGGTTTTTTAAACCTCACGCTACGCTCACTCACGCTACGCTCACTCACGCTACG
CTCACCTGCAGGAGAGGGTATATAATGGAAGCTCGAATTCCAGAAGCTTATACTCAGTG
CCCTGACTATATACTCAGTGCCCTGACTAT

Supplementary References

1. Kawano, F., Okazaki, R., Yazawa, M. & Sato, M. A photoactivatable Cre-loxP recombination system for optogenetic genome engineering. *Nat. Chem. Biol.* **12**, 1059-1064 (2016).
2. Shao, J. *et al.* Smartphone-controlled optogenetically engineered cells enable semiautomatic glucose homeostasis in diabetic mice. *Sci. Transl. Med.* **9**, eaal2298 (2017).
3. Taslimi, A. *et al.* Optimized second-generation CRY2-CIB dimerizers and photoactivatable Cre recombinase. *Nat. Chem. Biol.* **12**, 425-430 (2016).
